# Rotator Cuff Disorders: Practical Recommendations for Conservative Management Based on the Literature

**DOI:** 10.3390/medicina62020272

**Published:** 2026-01-27

**Authors:** Adrien J.-P. Schwitzguébel

**Affiliations:** Sports Medicine Division, Hôpital de la Providence, 2000 Neuchâtel, Switzerland; adrien.schwitzguebel@gmail.com

**Keywords:** shoulder, rotator cuff, classification, conservative management, non-surgical treatment, rotator cuff disorders, rehabilitation, musculoskeletal medicine, regenerative therapies, clinical protocol, sports medicine, return-to-play, clinical follow-up, regenerative medicine

## Abstract

Conservative management of rotator cuff disorders remains challenging, with no comprehensive, evidence-based framework integrating diagnosis, prognosis, rehabilitation, and biological therapies. Existing recommendations usually address isolated components of care, leading to inconsistent treatment strategies. This article proposes a global, pragmatic protocol for the non-surgical management of rotator cuff lesions, from initial assessment to long-term follow-up. Drawing on clinical expertise supported by recent literature, we outline a stepwise approach that begins with a comprehensive diagnostic process that combines history, clinical examination, and targeted imaging. Based on lesion type, associated shoulder or neurogenic conditions, and patient profile, rotator cuff disorders are stratified into three prognostic categories under conservative care: good, borderline, and poor prognosis, highlighting factors that require treatment adaptation or early surgical consideration. Rehabilitation objectives are structured around four domains: (1) inflammation and pain control, (2) mobility and scapular kinematics, (3) strengthening and motor control with tendon-sparing strategies, and (4) preservation or restoration of anatomy. For each prognostic category, we define a monitoring plan integrating clinical reassessment, ultrasound follow-up, and functional milestones, including return-to-play criteria for athletes. This comprehensive narrative review demonstrates that precise diagnosis and individualized rehabilitation can optimize medical follow-up, active strengthening, and complementary or regenerative therapies. Aligning therapeutic decisions with prognostic and functional goals allows clinicians to optimize patient satisfaction and recovery, providing a clear, evidence-informed roadmap for conservative management of rotator cuff disorders.

## 1. Introduction

Rotator cuff tears represent a common cause of shoulder pain and disability in adults [1]. Despite the growing body of literature on rotator cuff related disorders, there remains substantial heterogeneity across nonoperative rehabilitation programs and clinical follow-up strategies, which may contribute to variability in day-to-day clinical decision-making [2,3,4]. Nowadays, there is a need to help harmonize and standardize non-surgical management practices in outpatient settings and training environments specializing in Sports Medicine and Physical and Rehabilitation Medicine. In particular, existing guidelines rarely provide practical recommendations on longitudinal follow-up, imaging indications, or treatment adaptation, which can lead to variability in clinical decision-making. This narrative review is based on targeted literature searches and an experience-informed synthesis of the evidence, aiming to translate current concepts into a step-by-step clinician-oriented protocol.

Although it is widely accepted that exercise-based rehabilitation improves outcomes in rotator cuff disorders, the optimal characteristics of available rehabilitation programs remain insufficiently defined and largely clinician-dependent. While general rehabilitation principles are well described, a practical step-by-step recommendation is still lacking, supporting the need for clinician-oriented protocols. In this context, recent consensus statements, such as the Bern Consensus, which primarily targets athletes involved in shoulder demanding sports, provide useful overarching principles for rehabilitation and return to sport decision making, yet they remain relatively broad and do not translate into a rotator cuff specific, clinician oriented protocol for day-to-day management [5]. Concerning surgical decision, even if the decision-making is nowadays better documented, the decision between surgical and nonoperative management of rotator cuff tears remains complex and clinician-dependent [6], and must be balanced against the potential for tear progression over time [7], within an individualized, evidence-informed framework as outlined in current clinical practice guidelines [8].

More broadly, medical training and published recommendations often provide limited guidance on how to structure and individualize clinical follow-up over time, a competency that is frequently acquired through clinical experience rather than formal instruction. To date, step-by-step practical guidance remains scarce in the literature and is often replaced by broad conceptual principles and general decision-making recommendations.

The aim of this narrative review is to provide a clinically meaningful protocol to support clinicians in evidence-informed decision-making and up-to-date rehabilitation strategies in 2026.

## 2. Methods

This protocol is based on a narrative, experience-informed synthesis of the literature. The recommendations combine commonly accepted clinical practice with evidence-informed therapeutic principles. References were identified through targeted searches in PubMed using predefined keywords for each clinical situation, related to rotator cuff tears, tendinopathy, conservative treatment, rehabilitation, prognostic factors, and return-to-play. Google Scholar was used as a complementary search engine to identify additional relevant guidelines, consensus statements, and highly cited articles not retrieved through PubMed searches. Priority was given to systematic reviews, randomized controlled trials, and international recommendations, with preference for publications from the last 10 years unless older studies were considered landmark or essential for clinical context. The selection process was complemented by the author’s clinical expertise to ensure clinical applicability. This article does not follow a systematic review methodology and does not aim to provide an exhaustive analysis of all available studies.

## 3. Results

**Figure 1:**
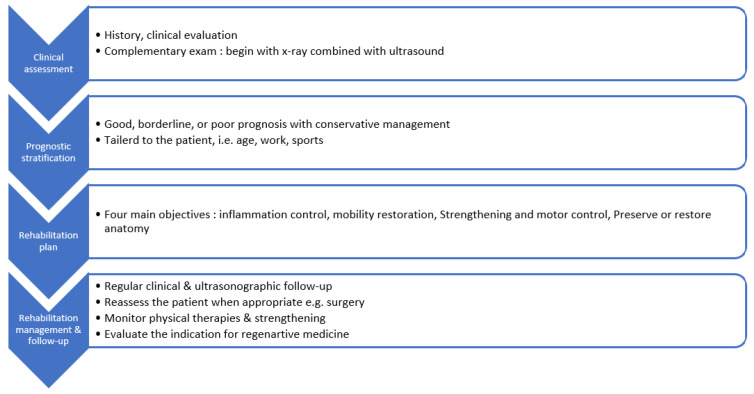
Stepwise clinical approach for the conservative management of rotator cuff disorders.

The stepwise clinical approach proposed in this protocol is summarized in Figure 1. The proposed approach integrates diagnostic clarification, prognostic stratification, structured rehabilitation objectives, and explicit follow-up pathways. It also describes the rational use of therapeutic options such as passive modalities, injectables, and regenerative procedures, all positioned as complementary tools that support structured active rehabilitation.

## 4. Discussion

Current guidelines on rotator cuff disorders primarily focus on diagnostic criteria, surgical indications, or general rehabilitation principles, but do not always provide detailed guidance on longitudinal clinical follow-up and treatment adaptation within conservative care. In contrast, the present protocol emphasizes a structured, stepwise approach integrating diagnostic clarification, prognostic stratification, rehabilitation objectives, and predefined reassessment points, allowing management to be adapted over time rather than applied as a static recommendation.

The literature on conservative management of rotator cuff disorders is abundant but remains heterogeneous. Available evidence primarily describes key rehabilitation concepts and consistently supports the effectiveness of active strengthening-based programs [2,3,4]. However, combined treatment strategies are less well described, and limited guidance exists regarding the optimal timing and integration of different interventions within a structured, longitudinal rehabilitation plan. While this flexibility allows individualization according to patient characteristics (e.g., sports participation and return-to-play demands), the step-by-step framework proposed in the present protocol may provide a clearer clinical roadmap for junior clinicians with limited experience, and may also encourage more experienced clinicians to critically integrate emerging rehabilitation techniques into their treatment planning.

Surgical decision-making in rotator cuff disorders remains complex and controversial, and cannot be reduced to tear size or imaging findings alone [6]. In particular, the classification of partial-thickness tears exceeding 50% as a borderline category is debated, as many surgeons consider this threshold a surgical indication. However, several studies have demonstrated that conservative management can still lead to favorable clinical outcomes in selected patients, including those with larger partial tears [8,9]. Accordingly, this protocol deliberately classifies such lesions as borderline to promote individualized decision-making and to encourage reassessment based on symptom evolution and functional recovery rather than reliance on a strict morphological threshold. Tear progression is also frequently cited as a key argument to support early surgical referral [8], yet the available evidence remains difficult to interpret in routine practice due to marked heterogeneity in the definitions of progression and in reported progression rates. A recent systematic review highlighted substantial disparities across studies in how progression is defined, measured, and reported, resulting in wide variations in estimated progression rates [7]. In this context, structured conservative pathways with predefined reassessment points may facilitate timely treatment adaptation and escalation, allowing referral decisions to be guided by symptom evolution, function, and objective clinical changes rather than by imaging-based assumptions alone [8].

The proposed follow-up strategy is designed to support early active rehabilitation and, when appropriate, an early return to sport, which is often requested by athletes, while maintaining clinical safety through predefined reassessment points [10]. In selected cases, imaging can be integrated as an adjunct to clinical monitoring to document tendon status when symptoms or function evolve, or when an objective reassessment is required before progressing rehabilitation intensity [11]. Moreover, delayed follow-up imaging may help detect early worsening or progression, potentially preventing complications, and may also help to document a reduction in direct signs of tendon pathology, such as tendinosis features or Doppler hypervascularity, as well as indirect signs, such as subacromial bursitis, which may support treatment response and reinforce the decision to advance to the next rehabilitation phase. Importantly, imaging findings should not be interpreted in isolation, but rather integrated with symptom evolution, functional recovery, and load tolerance.

Another distinctive feature of this protocol is the explicit positioning of active rehabilitation as the cornerstone of conservative management, while passive modalities, injectable treatments, and regenerative approaches are framed as adjunctive options. This integration aims to promote a rational and proportionate use of available therapeutic tools while maintaining clinical coherence. Rehabilitation progression is deliberately criteria-based rather than time-based, with advancement guided by clinical and functional recovery, symptom evolution, and tolerance to load, in order to preserve clinical judgment and reflect real-world conservative practice.

A key strength of the present protocol is its longitudinal and structured follow-up strategy, which translates broad rehabilitation concepts into a practical clinical pathway. The protocol includes predefined decision nodes and reassessment points to guide treatment adaptation over time, rather than applying conservative management as a static recommendation. Rehabilitation progression is deliberately criteria-based rather than strictly time-based, allowing advancement to be driven by symptom evolution, functional recovery, and load tolerance. Finally, the protocol integrates adjunctive therapies within a rational hierarchy, emphasizing active rehabilitation as the cornerstone of care while framing passive modalities and injectable or regenerative options as supportive interventions when clinically indicated.

## 5. Limitations

This work has several limitations. First, it is a narrative review and does not follow a systematic review methodology, therefore reference selection may be subject to selection bias and does not aim to provide an exhaustive analysis of all available evidence. Second, the protocol was not developed through a formal Delphi process or multidisciplinary consensus panel, and it reflects a clinician-oriented synthesis rather than a consensus-based guideline. Third, the proposed pathway has not been prospectively validated, and its impact on clinical outcomes, return-to-sport trajectories, and healthcare utilization remains to be established. Future research should include implementation studies and prospective validation in other outpatient settings, ideally with multicenter designs, assessment of inter-clinician reproducibility, and cost-effectiveness analyses. Finally, the protocol may require adaptation depending on local resources and healthcare systems, including imaging availability, access to specialized rehabilitation, and the specific demands of different sports and patient populations.

## 6. Conclusions

This narrative review proposes a pragmatic and structured approach to conservative management of rotator cuff disorders, emphasizing individualized care, active rehabilitation, and rational use of adjunct therapies. By translating available evidence and clinical experience into a coherent and usable protocol, it aims to support consistent clinical decision-making, longitudinal follow-up, and structured progression of rehabilitation in everyday practice.

**EVALUATION**

**1.**  **Patient history and clinical examination**
  a. History: onset and mechanism of injury (traumatic versus degenerative), and evolution or fluctuation of symptoms over time.
  b. Differential diagnosis: Consider neurogenic, articular, and referred pain sources in the differential diagnosis.
  c. Clinical examination: Clinical examination includes assessment of range of motion, strength testing, and specific maneuvers for impingement and rotator cuff integrity.
**2.**  **Initial imaging assessment**

**a.**  **Standard radiography:**
  i. Should be systematically proposed, particularly to assist in differential diagnosis. To include true anteroposterior views in three rotations and a Neer view.
  ii. To add an axial view if glenohumeral osteoarthritis is suspected.
  iii. To add axial and Bernageau views if glenohumeral instability is suspected.
  iv. To add a scapular Y-view if acromioclavicular joint involvement is suspected.
**b.**  **Ultrasound:**
  i. To perform systematically as a first-line imaging modality, ensuring complete and comprehensive assessment [12,13].
  ii. To include dynamic evaluation for impingement mechanisms, biceps tendon instability, and friction phenomena [14,15].
  iii. To use contralateral comparison and color or power Doppler (with the patient supine to enhance sensitivity) to assess vascularization and inflammatory activity [14].
**c.**  **MRI**
  i. Indicated when surgical management is being considered; MR arthrography may be considered in selected cases.
  ii. Also indicated when diagnosis remains uncertain after radiography and ultrasound.
**d.**  **CT or MR arthrogram**
  i. To determine whether a cuff lesion is full-thickness [16].
  ii. To evaluate associated intra-articular pathology, such as labral lesions, instability, or osteoarthritis.
**e.**  **CT scan or SPECT-CT**
  i. To be reserved for exceptional cases (bone pathology or complex/unclear diagnosis).
  ii. To help differentiate mechanical from metabolic pain sources, particularly in cases of therapeutic failure [17].

**DIAGNOSIS**

**1.**  **Rotator cuff lesion (Table 1** **)**

**a.**  **Good prognosis with conservative management**
  i. Tendinopathies without tear or with interstitial tear [18].
  ii. Small acute traumatic tears involving less than 50% of tendon thickness in any plane [19,20,21].
  iii. Calcific tendinopathies [22].
  iv. Acute isolated rupture of the long head of the biceps tendon [23].
**b.**  **Borderline prognosis with conservative management**
  i. Tendon tears (bursal-, articular-, or full-thickness) occurring without tendon retraction or muscle atrophy [9].
  ii. Acute traumatic partial tears affecting ≥ 50% of tendon thickness in any plane, excluding the long head of the biceps [9].
  iii. Tendon tears showing retraction or muscle atrophy in elderly or low-demand patients [24].
  iv. Suspected instability of the long head of the biceps with pulley lesion [25].
  v. Post-fracture status of the proximal humerus with residual displacement, increasing the risk of secondary impingement.
**c.**  **Poor prognosis with conservative management**
  i. Complete or high-grade partial tears associated with retraction or muscle atrophy, particularly in younger and active patients.
  ii. Instability of the long head of the biceps combined with a contiguous tear of the supraspinatus or superior subscapularis tendon [25].
  iii. Complete acute ruptures other than those involving the long head of the biceps [26].

2. **Associated factors**

**a.**  **Impingement syndromes**
  i. Subacromial impingement
  ii. Posterosuperior impingement
  iii. Anterior impingement
**b.**  **Glenohumeral disorders**
  i. Adhesive capsulitis
  ii. Labral lesion (SLAP)
  iii. Glenohumeral osteoarthritis
  iv. Glenohumeral instability
**c.**  **Acromioclavicular joint**
  i. Arthropathy
  ii. Dislocation
**d.**  **Neurogenic conditions**
  i. Cervicogenic brachialgia
  ii. Thoracic outlet syndrome
  iii. Focal neuropathies (e.g., suprascapular)
  iv. Parsonage-Turner syndrome
  v. Axillary nerve entrapment

**REHABILITATION OBJECTIVES (Table 2)**

**Table 2:** Overview of rehabilitation objectives.

Objective	Primary Goal	Key Interventions or Strategies
#1—Inflammation control	To reduce pain and local inflammation in order to enable early rehabilitation	— Short course of anti-inflammatory drugs — Neuromodulation or dry needling when appropriate
#2—Mobility restoration	To recover full, pain-free range of motion and prevent joint stiffness	— Early self-mobilization and stretching — Adhesive capsulitis management if applicable — Postural and scapular-control reeducation
#3—Strengthening and motor control	To rebuild tendon and muscle function while optimizing dynamic stability	— Progressive and tendon-sparing strengthening — Scapular and kinetic-chain integration — Functional or sport-specific retraining
#4—Preserve or restore anatomy	To support tendon healing and tissue homeostasis	— Nutritional optimization — Management of calcific tendinopathy — Regenerative interventions considered once impingement has been corrected

**1.**  **Inflammation**

a. To manage inflammatory processes, bursitis, and joint effusion effectively.
b. To ensure adequate pain control and patient comfort.
c. To monitor for potential nerve irritation or early signs of complex regional pain syndrome (CRPS).

**2.**  **Mobility**

a. To maintain or restore full joint range of motion.
b. To optimize scapular kinematics and address impingement mechanisms when present.
c. To enhance proprioceptive function and promote coordinated scapulothoracic and glenohumeral motion control.

**3.**  **Strengthening**

a. To improve muscular strength and global functional performance.
b. To implement tendon-sparing strengthening protocols, particularly for the supraspinatus.
c. To control laxity and dynamic instability while minimizing the risk of recurrent microtrauma.

**4.**  **Preserve or restore anatomy**

a. To promote tissue healing and biological stimulation within the repair process.
b. To support neural recovery and neuromuscular reintegration when applicable.

**REHABILITATION MANAGEMENT**

**1.**  **Surgical decision making**

**a.**  **Indications for early surgery**
  i. Acute traumatic partial tears involving more than 50% of tendon thickness, or complete ruptures, particularly in young and active patients.
  ii. Any lesion classified as poor prognosis with conservative management. Notably retracted tears, early fatty atrophy, or complex biceps pulley lesions in young and active individuals [27].
**b.**  **Indications for delayed or secondary surgery**
  i. Lesions with borderline prognosis under conservative management that remain symptomatic after an adequate rehabilitation program (minimum 12 weeks including progressive active strengthening) [28].
  ii. Degenerative tendon lesions in young and active patients: conservative management remains the first-line option, with surgery considered only in case of treatment failure [27,29].
  iii. Acute small (<50%) tendon ruptures: conservative and surgical approaches generally provide comparable outcomes; surgery may remain optional [30].
  iv. Full-thickness tears in middle-aged or elderly patients: favorable outcomes are often achievable through non-operative management [24].
**c.**  **Contraindications or deferral of surgery**
  i. Adhesive capsulitis: prioritize restoration of range of motion before considering surgical intervention; surgery should be reconsidered only once stiffness has resolved [31,32].
  ii. Neurogenic etiologies (e.g., cervical radiculopathy, suprascapular neuropathy, Parsonage–Turner syndrome): manage the neurological condition first, then reassess the indication for surgery.

**2.**  **Conservative management: physician-guided monitoring plan (Table 3)**

**a.**  **Clinical follow-up**
  i. Schedule clinical reassessments every 6–12 weeks, depending on the patient’s progression and activity level.
  ii. Consider earlier follow-up (2–4 weeks) in acute cases or in high-demand athletes to optimize load management and pain control.
  iii. At each visit, monitor pain (VAS), function (SANE or Constant score), and activity tolerance [31].
**b.**  **Ultrasound monitoring**
  i. Perform ultrasonographic follow-up as part of the routine clinical evaluation to monitor tendon integrity, bursal inflammation, and impingement dynamics [11,21].
  ii. Stability or gradual improvement in echotexture and vascularization, even without complete anatomic healing, generally reflects a favorable response to conservative treatment.
  iii. Progressive tear enlargement or persistent bursal hyperemia may justify reconsidering either conservative or surgical management.
**c.**  **Multidisciplinary reassessment**
  i. Reassess the indication for surgery when pain, weakness, or loss of function persist despite at least 12 weeks of appropriate, structured rehabilitation (see above).
  ii. Surgical reconsideration should also be discussed if imaging reveals progressive tendon retraction, atrophy, or loss of function despite good compliance [9,24].
  iii. Consider rheumatologic evaluation when inflammatory or systemic factors are suspected, such as atypical pain distribution, morning stiffness, or persistent synovial hypervascularization on ultrasound.
**d.**  **Return-to-play decision**
  i. Return to play should be gradual and supervised by a sports physician or physiotherapist experienced in shoulder rehabilitation, with progressive workload monitoring.
  ii. Presence of mild to moderate pain showing progressive improvement over time is acceptable for return-to-play progression [10,33].
  iii. Ensure absence of dynamic impingement or tear progression on follow-up ultrasound [34,35].
  iv. Confirm sufficient strength and functional performance to execute sport-specific tasks without compensatory mechanisms, for example using a limb symmetry index ≥ 90% compared with the contralateral side [10,33].

**3.**  **Objective #1: Inflammation control**

**a.**  **Analgesics and NSAIDs**
  i. Use analgesics and short courses of nonsteroidal anti-inflammatory drugs (NSAIDs) to manage acute pain and reduce inflammatory response.
  ii. Prolonged or repeated use should be avoided, as it provides no proven benefit for tendon healing and may exert detrimental effects on tendon biology [36,37].
**b.**  **Corticosteroid injections (with or without hyaluronic acid)**
  i. Corticosteroid injections may be considered for short-term pain control when pain significantly limits rehabilitation.
  ii. Subacromial injections are indicated for bursitis, superficial or transfixing tendinosis, or subacromial impingement.
  iii. Glenohumeral injections are indicated for articular-sided tendinosis, labral pathology, or glenohumeral osteoarthritis.
  iv. Acromioclavicular injections are indicated for acromioclavicular joint involvement.
  v. Combination with hyaluronic acid may enhance short-term pain relief and joint mobility, and may contribute to tendon preservation [38].
  vi. Injections should be limited to single administrations or short series to minimize potential catabolic effects on tendon tissue and reduce the long-term risk of surgical conversion [39,40,41].
**c.**  **Neurogenic pain management**
  i. Consider targeted management of neurogenic pain when clinical features suggest a neuropathic component, either as a primary or associated condition.
  ii. Assessment may include diagnostic nerve blocks and/or electromyographic studies when appropriate and available.
**d.**  **Physical therapies in the early subacute phase**
  i. Manual therapy, gentle joint mobilizations, soft-tissue techniques, and dry needling can be initiated early in the subacute phase to reduce residual muscle tension, promote pain modulation, and facilitate recovery of shoulder mobility [42].

**4.**  **Objective #2: mobility**

**a.**  **General mobility principles**
  i. Maintain periscapular balance and glenohumeral articular amplitudes, especially to prevent secondary impingement [43].
  ii. Minimize shear forces on the tendon whenever possible [44].
  iii. Consider supportive taping or bracing for short-term proprioceptive feedback or pain relief [45,46].
**b.**  **Limitation of range of motion and adhesive capsulitis**
  i. Focus early, controlled, and progressive rehabilitation on self-mobilization exercises [47].
  ii. Consider capsular distension [48], suprascapular nerve block [49], with or without manipulation under appropriate anesthesia [50].
**c.**  **Impingements**
  i. Emphasize scapular opening, postural correction, and pectoralis minor stretching [43].
  ii. Use sleeper stretch in cases of posterosuperior impingement [51].

**5.**  **Objective #3: strengthening (Table 4)**

**a.**  **Early phase**
  i. Initiate a self-directed exercise program aiming to maintain neuromuscular activation and preserve muscle capacity [43,52].
  ii. Implement hypertrophic strengthening protocols [53] with minimal inflammatory impact, such as blood flow restriction training [54,55] or isokinetic strengthening [56].
  iii. Reinforce periscapular and rotator cuff muscles through progressive loading, including high-load eccentric exercises [57], to enhance dynamic stability and promote tendon remodeling.
  iv. In cases of supraspinatus tear, apply relative supraspinatus-sparing strategies, focusing on external rotators, adductors, and deltoid strengthening to support shoulder abduction and humeral head centering [58].
**b.**  **Late phase**
  i. Perform objective strength testing to guide load progression and confirm readiness for higher functional demands [59].
  ii. Integrate kinetic-chain coordination once local control and strength are adequately restored, ensuring efficient transfer to complex and multiplanar movements [60].
  iii. Progress toward sport-specific and functional exercises emphasizing movement efficiency, endurance, and controlled power generation [61,62].

**6.**  **Objective #4: preserve or restore anatomy**

**a.**  **Trophism & nutrition**
  i. Beyond mechanical stimulation, nutritional and biological strategies can further support tendon healing and tissue homeostasis.
  ii. Ensure adequate daily protein intake (1.2–1.6 g/kg body weight) to support overall tissue recovery, including muscle maintenance and connective tissue remodeling. Carbohydrate intake should also be sufficient to maintain energy availability and prevent catabolic states during rehabilitation. [63,64].
  iii. Consider nutritional supplements that may support tendon and muscle healing, particularly collagen peptides [65], vitamins C and D [66], and omega-3 fatty acids (EPA/DHA) [66,67].
  iv. Address modifiable risk factors for tendinopathies, including exposure to quinolones or corticosteroids [68], underlying rheumatologic or autoimmune diseases, and possibly menopause (considering hormone replacement therapy) [69]. Potential dental issues may also be relevant, although current evidence remains very limited [70].
**b.**  **Calcic tendinopathy**
  i. Calcifications should be removed or reduced when feasible.
  ii. Consider ultrasound-guided needle aspiration when a single, swollen calcification is present [22].
  iii. Focused shockwave therapy is indicated when calcifications are small, multiple, or not amenable to needle aspiration [71,72].
**c.**  **Regenerative medicine (Table 5)**
  i. Regenerative medicine should be considered with caution and only after effective management of pre-existing impingements and optimization of mechanical load and rehabilitation.
  ii. Realistic clinical indications include chronic tendinopathy or small to moderate, non-retracted partial tears.
  iii. Platelet-rich plasma (PRP) ± tendon needling, despite conflicting evidence, may promote tendon healing in selected cases [73,74,75].
  iv. Focused shockwave therapy can provide additional biological stimulation, although clinical responses are generally moderate [71,76,77].
  v. Emerging adjuncts, sometimes combined with PRP, include matrix components such as injectable collagen or viscosupplementation [38,78].
  vi. Potentially more potent but technically demanding approaches involve stromal vascular fraction or nanofat injections; these remain strictly experimental [79].
  vii. A variety of other passive or device-based regenerative modalities, including hyperbaric oxygen, electrolysis, low-level laser therapy, and TECAR, have also been proposed. Their clinical relevance remains uncertain, and structured active rehabilitation should remain the foundation of treatment before considering such complementary options [80].

## Figures and Tables

**Table 1:** Prognostic stratification of rotator cuff lesions under conservative management.

Prognostic Category	Typical Lesion Types	Key Characteristics and Associated Findings
Good prognosis	— Tendinopaty without tear or with interstitial tear — Small traumatic tear (<50% tendon thickness) — Calcific tendinopathy — Isolated rupture of the long head of the biceps	— No tendon retraction — Preserved muscle trophism — Limited structural damage
Borderline prognosis	— Partial-thickness tear ≥ 50% — Non-retracted full-thickness tear — Pulley lesion with biceps instability — Post-fracture proximal humerus with residual displacement	— Moderate structural compromise — None or limited fatty infiltration — Possible pain persistence — Function usually preserved
Poor prognosis	— Full-thickness tear with retraction or muscle atrophy (young or high-demand patient) — Combined biceps instability with supraspinatus or subscapularis tear — Large acute tear (young or high-demand patient)	— Retraction and poor tendon quality — Advanced muscle atrophy — High functional demand — Low potential for spontaneous recovery

**Table 3:** Clinical and imaging monitoring plan during conservative management.

Phase	Clinical Focus	Imaging Focus (Ultrasound)	Reassessment/Criteria
Initial phase	— Control pain and inflammation — Optimize posture and load — Maintain gentle mobility	— Baseline evaluation of tendon integrity and vascularity — Identify dynamic impingement mechanisms	— Review every 2–3 weeks or as clinically indicated — Baseline ultrasound if not yet performed
Functional recovery phase	— Restore full range of motion and scapular control — Initiate isometric and low-load strengthening	— Monitor bursitis and vascular hyperemia — Verify absence of tear progression	— Clinical and ultrasound follow-up every 3–6 weeks depending on symptoms
Strength and control phase	— Progressive strengthening (BFR, isokinetic, eccentric) — Integrate kinetic-chain and proprioceptive work	— Assess echotexture improvement and tendon stability	— Reassess approximately every 6 weeks or according to progression
Return-to-activity phase	— Consolidate strength, proprioception, and endurance — Prepare for sport- or work-specific demands	— Confirm tendon remodeling and absence of dynamic impingement	— Reassess every 6–8 weeks — Return-to-play criteria: pain-free, symmetric function, stable imaging

**Table 4:** Progressive strengthening framework.

Rehabilitation Phase	Clinical Objectives and Focus	Key Exercises/Strategies
Initial phase (after pain control)	Restore neuromuscular activation and maintain baseline muscle trophism. Emphasize scapular stability and gentle rotator cuff recruitment without overload.	— Isometric and low-load activation — Blood flow restriction (BFR) or light isokinetic work — Postural correction and proprioceptive retraining
Intermediate phase	Rebuild tendon capacity and dynamic stability through progressive loading. Promote tendon remodeling and endurance.	— Gradual strengthening including isokinetic and eccentric exercises — Scapular-control and closed-chain drills — Core and kinetic-chain integration
Advanced phase	Optimize movement efficiency and restore functional control. Ensure symmetrical coordination and controlled power generation.	— High-load multimodal training — Multiplanar strength exercises — Controlled endurance and power development
Return-to-activity phase	Transition safely to sport or occupational activities. Maintain gains and prevent recurrence through optimized kinetic-chain control.	— Functional testing and monitored load progression — Sport- or task-specific retraining — Preventive maintenance program

**Table 5:** Summary of regenerative and biological therapies.

Therapy	Clinical Indications	Main Therapeutic Effects	Evidence Level and Comments
1. Platelet-rich plasma (PRP) ± tendon needling	Chronic tendinopathy or small to moderate, non-retracted partial tears after correction of mechanical impingement	— Pain control — Support tendon remodeling	Evidence remains conflicting. Protocols, leukocyte content, and dosing vary widely. Clinical benefits are inconsistent; anti-inflammatory and analgesic effects remain debated.
2. Focused shockwave therapy	Chronic tendinopathy or calcific tendinopathy	— Anti-inflammatory effects — Pain modulation — Promote remodeling	Evidence is moderate and protocol-dependent. Best used as an adjunct to rehabilitation. Experimental studies support anti-inflammatory and neoangiogenic effects.
3. Injectable matrix components (collagen, viscosupplementation)	Chronic tendinopathy or partial tear; may be combined with PRP	— Pain modulation — Support extracellular matrix remodeling	Evidence is limited and emerging. May improve viscoelastic properties and matrix organization; clinical impact variable.
4. Stromal vascular fraction (SVF) or nanofat injection	Chronic or degenerative lesions refractory to standard care	— Anti-inflammatory effects — Promote remodeling and trophic support	Experimental therapy with limited early clinical evidence. Technically demanding procedures with promising biological rationale, requiring further clinical validation.
5. Other passive or device-based modalities (hyperbaric oxygen, electrolysis, low-level laser therapy, TECAR, etc.)		— Pain modulation — Uncertain/variable effects	Evidence remains weak or inconsistent. Mechanistic rationale often speculative. Should not replace structured active rehabilitation.

## Data Availability

No new data were created or analyzed in this study.
